# A ten-year retrospective analysis of decompressive craniectomy or craniotomy after severe brain injury and its implications for donation after brain death

**DOI:** 10.1038/s41598-024-66129-3

**Published:** 2024-07-02

**Authors:** Jan Sönke Englbrecht, Charis Bajohr, Alexander Zarbock, Walter Stummer, Markus Holling

**Affiliations:** 1 Department of Anesthesiology, Intensive Care Medicine and Pain Therapy, University Hospital Münster, Albert-Schweitzer-Campus 1, Building A1, 48149 Münster, Germany; 2 Department of Anesthesiology, Herz-Jesu-Hospital Münster-Hiltrup, Münster, Germany; 3 Department of Neurosurgery, University Hospital Münster, Münster, Germany

**Keywords:** Brain injury, Brain death, Craniotomy, Decompressive craniectomy, Intracranial pressure, Organ donation, Cell death in the nervous system, Diseases of the nervous system, Brain injuries

## Abstract

Craniotomy or decompressive craniectomy are among the therapeutic options to prevent or treat secondary damage after severe brain injury. The choice of procedure depends, among other things, on the type and severity of the initial injury. It remains controversial whether both procedures influence the neurological outcome differently. Thus, estimating the risk of brain herniation and death and consequently potential organ donation remains difficult. All patients at the University Hospital Münster for whom an isolated craniotomy or decompressive craniectomy was performed as a treatment after severe brain injury between 2013 and 2022 were retrospectively included. Proportion of survivors and deceased were evaluated. Deceased were further analyzed regarding anticoagulants, comorbidities, type of brain injury, potential and utilized donation after brain death. 595 patients were identified, 296 patients survived, and 299 deceased. Proportion of decompressive craniectomy was higher than craniotomy in survivors (89% vs. 11%, p < 0.001). Brain death was diagnosed in 12 deceased and 10 donations were utilized. Utilized donations were comparable after both procedures (5% vs. 2%, p = 0.194). Preserved brain stem reflexes as a reason against donation did not differ between decompressive craniectomy or craniotomy (32% vs. 29%, p = 0.470). Patients with severe brain injury were more likely to survive after decompressive craniectomy than craniotomy. Among the deceased, potential and utilized donations did not differ between both procedures. This suggests that brain death can occur independent of the previous neurosurgical procedure and that organ donation should always be considered in end-of-life decisions for patients with a fatal prognosis.

## Introduction

The treatment of patients with severe brain injury (BI) is based on the central concept that the prevention of secondary injuries is associated with better clinical outcomes. Increased intracranial pressure (ICP) and reduced cerebral perfusion pressure (CPP) are important causes of secondary brain injury that are associated with worsened outcomes^1^. These secondary processes escalate brain edema after severe BI and lead to increased ICP, which, in turn, causes a reduction in CPP and cerebral blood flow. This causes additional brain edema, forming a ‘vicious circle’ that can potentially worsen outcome and eventually lead to brain herniation and death^1^. Modern neurocritical care management incorporates tiered ICP- and CPP-guided strategies that include both medical and surgical interventions. The neurosurgical treatment to prevent consequential damage after severe BI can be categorized into two distinct types based on the removal of a bone flap: craniotomy (CO), as a surgical approach to remove a mass lesion (e.g. hematoma evacuation), and where the bone flap is replaced and decompressive craniectomy (DC), where craniotomy and durotomy represent the therapeutic measure and the bone flap is left out^2,3^. There is limited evidence with respect to the added value of performing a DC to improve patient outcomes^3,4^. Some studies suggest that DC has the potential to reduce mortality after severe BI^5–10^, although this is not consistently found for all types of BI, and DC can be associated with a higher risk of unfavorable outcomes^3,11–13^. A non-randomized cohort study demonstrated a lower mortality rate in patients undergoing primary DC compared to CO after evacuation of an acute subdural hematoma^14^, whereas others found no differences in outcomes^3,11^. Thus, it can be challenging to predict which individual patient will benefit from DC or CO^15,16^. Nevertheless, frequency of DC increased over the last years^17^. This could lead to more patients surviving after severe BI, as brain herniation and death is prevented. Consequently, it can be hypothesized that the incidence of brain death (BD) decreases with an increase in DC compared to other therapeutic options^17,18^. As the determination of BD is a mandatory requirement for post-mortem organ donation (donation after brain death (DBD)) in many countries, including Germany, this could lead to the assumption that the number of DBD decreases with increasing DC^17^.

This aspect is rarely discussed in the literature. Although death is an considered outcome criterion in studies about DC and CO after severe BI, to our knowledge, most studies did not define potential BD as a separate outcome criteria^2,3,8,9,11,14^. One trial reported brain herniation as the reason for death in one of 20 patients with DC after malignant middle cerebral artery infarction^6^. Two older studies found that DC was performed with similar frequency in donors and non-donors and that DC had no influence on the progression to BD after severe BI, suggesting no association between DC and conversion to donor status^18,19^. Schulte et al. postulated that despite an increasing number of DC in Germany in a period from 2010 to 2016, utilized donations should not decrease. However, they did not analyze the potential influence of DC on the incidence of BD but they detected a parallel increase in the total numbers of possible donors in the same period and suggested that this results in a constant number of possible donors^17^. Overall, differences in estimated DBD after severe BI cannot be satisfactorily explained yet due to an obvious lack of evidence^20^. Whether DC or CO have a different influence on the number of potential and utilized DBD (defined as a person whose clinical condition is suspected to fulfill brain death criteria and an actual donor from whom at least one organ was transplanted, respectively^21^) thus remains questionable. Patients after DC are usually not regarded to qualify for BD as ICP is not assumed to reach levels critical enough to cause cerebral perfusion failure, leading to the assumption, that DBD is less likely after DC^17,22^. In addition, diagnosis of BD may be more challenging, as the reliability to demonstrate cerebral circulatory arrest, as a prerequisite to determine BD, can be affected in patients after DC^23–26^.

The present study aimed to analyze patients who underwent CO or DC to prevent or treat consequential damage after severe BI. We sought to answer whether the number of potential and utilized DBD differed by DC or CO to aid critical care staff in estimating the potential of DBD as part of an end-of-life concept. We hypothesize that the number of potential and utilized DBD is lower in deceased after DC compared to CO.

## Results

**Figure 1:**
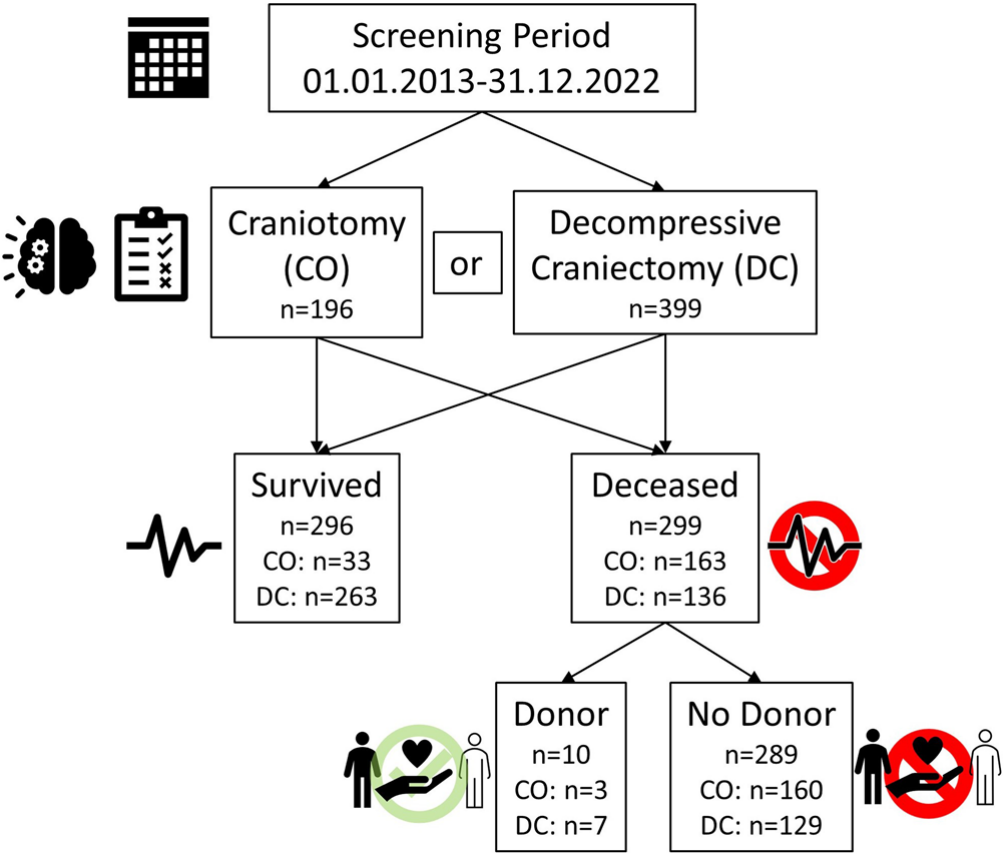
Study flowchart. *CO* craniotomy, *DC* decompressive craniectomy.

595 patients (mean age: 55.9 ± 19.7) who underwent CO or DC after severe BI were identified between 2013 and 2022 (Fig. 1). DC was performed in 399 and CO in 196 patients, respectively. Patients with DC were significantly younger than patients with CO (Table 1).

**Table 1:** Demographics of the study cohort.

	n [mean age ± SD]	male/female	CO [mean age ± SD]	DC [mean age ± SD]
Included patients	595 [55.9 ± 19.7]	355/240	196 [58.6 ± 20.9]*	399 [54.6 ± 19.0]*
Survivors	296 [52.2 ± 19.5]^†^	188/108	33 [41.9 ± 20.6]**	263 [53.5 ± 19.0]**
Deceased	299 [59.6 ± 19.3]^†^	167/132	163 [62.0 ± 19.3]***	136 [56.7 ± 19.0]***
Diagnosis of BD initiated	16 [29.1 ± 15.0]	8/8	5	11
BD confirmed	12 [24.5 ± 14.6]	7/5	4	8
Utilized Donor	10 [26.1 ± 15.5]^‡^	5/5	3 [21.3 ± 7.4]	7 [28.1 ± 16.4]

*SD* standard deviation, *CO* craniotomy, *DC* decompressive craniectomy, *BD* brain death. ^†^p < 0.001 (age of deceased vs. survivors); ^‡^p < 0.001 (age of deceased donors vs. deceased without donation); *p = 0.020 (age of all patients with DC vs. CO); **p = 0.001 (age of survivors with DC vs. CO); ***p = 0.018 (age of deceased with DC vs. CO).

Of all identified patients, 296 (49.7%) survived. DC was performed in 263 (89%) and CO in 33 (11%) of the survivors. The remaining 299 (50.3%) patients died during their hospital stay, with DC in 136 (45%) and CO in 163 (55%) of the deceased. Diagnosis of BD was initiated in 16 deceased and confirmed in 12 by ancillary testing (4% of all deceased), whereas the remaining four cases (three after DC and one after CO) showed preserved brain stem reflexes (failed apnea testing) during BD diagnostic. In two cases with confirmed BD, consent to donation was refused, resulting in 10 utilized DBD in our cohort (3% of all deceased). Donors were significantly younger than the deceased without donation (Table 1).

### Length of stay, anticoagulant medication and comorbidities in deceased patients

Mean length of stay of all deceased on the intensive care unit was 294 h. One third of the patients were taking anticoagulants at the time of hospital admission. Arterial hypertension was the most frequent comorbidity, followed by atrial fibrillation and arteriosclerosis (peripheral, coronary or cerebral, Table 2). A one-way MANOVA showed no statistically significant difference between the independent variables (DC or CO) on the combined dependent variables (length of stay, anticoagulant medication and comorbidities; F [13, 285]  = 1.116, p < 0.345, partial η^2^ = 0.048, Wilk’s Λ = 0.952).

**Table 2:** Prevalence of anticoagulatory medication and comorbidities in the deceased.

	Total	DC	CO
Patients [n]	299	136	163
LOS [h, mean ± SD]	294 h ± 459 h	279 h ± 375 h	307 h ± 520 h
Anticoagulatory medication			
DOAC or VKA	59 [20%]	19 [14%]	40 [25%]
Antiplatelet therapy	40 [13%]	16 [12%]	24 [15%]
Comorbidities			
Arterial hypertension	60 [20%]	22 [16%]	38 [23%]
Atrial fibrillation	47 [16%]	17 [13%]	30 [18%]
PAD, CSVD or CAD	49 [16%]	22 [16%]	27 [17%]
Solid malignancies	26 [9%]	10 [7%]	16 [10%]
Leukemia or lymphoma	20 [7%]	9 [7%]	11 [7%]
Chronic kidney disease	13 [4%]	5 [4%]	8 [5%]
Heart failure (NYHA ≥ 3)	6 [2.0%]	2 [1.5%]	4 [2.5%]
Liver cirrhosis	5 [1.7%]	2 [1.5%]	3 [1.8%]
Thromboembolic disease	3 [1.0%]	2 [1.5%]	1 [0.6%]

*DC* decompressive craniectomy, *CO* craniotomy, *LOS* length of stay, *DOAC* direct oral anticoagulants, *VKA* vitamin K antagonists, *PAD* peripheral artery disease, *CSVD* cerebral small vessel disease, *CAD* coronary artery disease.

### Type of brain damage in deceased patients

The most frequent reason for severe BI in the deceased was traumatic brain injury (TBI, 32%), followed by acute ischemic stroke (AIS, 27%) and spontaneous intracerebral hemorrhage (ICH, 20%), respectively. Proportion of DC was highest in patients with anoxic BI after cardiac arrest (60%), followed by AIS (57%), non-traumatic subarachnoid hemorrhage (SAH, 48%) and ICH (45%), respectively (Table 3).

**Table 3:** Type of brain injury and evaluation of potential organ donation.

Type of BI in deceased (n = 299)	n (%)	DC, n (%)	Age [mean ± SD]	Utilized DBD
Traumatic brain injury	95 (32)	37 (39)	60.6 ± 23.2	6
Acute ischemic stroke	82 (27)	47 (57)	62.5 ± 12.7	0
Intracranial hemorrhage	60 (20)	27 (45)	61.1 ± 19.4	1
Brain tumor	26 (9)	8 (31)	55.4 ± 21.4	0
Subarachnoid hemorrhage	21 (7)	10 (48)	55.0 ± 12.5	1
CNS-infection	10 (3)	4 (40)	46.2 ± 24.9	1
Anoxic BI after cardiac arrest	5 (2)	3 (60)	41.8 ± 5.2	1
Reason against DBD in deceased				
Refused consent	112 (37)	48 (43)	58.0 ± 19.9	
Preserved brain stem reflexes or inconclusive diagnostic of BD	90 (30)	43 (48)	65.6 ± 13.7	
Medical contraindication	51 (17)	23 (45)	51.0 ± 18.1	
Therapeutic limitations due to patients will	33 (11)	13 (39)	73.5 ± 11.3	

*BI* brain injury, *DC* decompressive craniectomy, *SD* standard deviation, *DBD* donation after brain death, *CNS* central nervous system.

### Reasons against donation in deceased patients

Refused consent was the predominant reason for a donation not utilized (112 cases, 37%). In 86 cases, a complete loss of all brainstem reflexes was not detectable during hospitalization, so that the formal BD diagnostic was not initiated at all. In four additional cases, BD diagnostic was initiated, but the formal examination revealed preserved brainstem reflexes (preserved spontaneous breathing), so that in a total of 90 cases (30%) preserved brainstem reflexes were the reason against donation. The medical condition of the patient was the reason against donation in 51 cases (17%) (Table 3).

### Influence of DC and CO on outcome parameters

Proportion of DC was significantly higher in survivors than in deceased (89% vs. 45%, p < 0.001), whereas the number of utilized DBD was not significantly different between deceased after DC or CO (5% vs. 2%, p = 0.194). The incidence of preserved brain stem reflexes/inconclusive diagnostic of BD as a reason for a donation not utilized was not different between deceased after DC or CO (33% vs. 29%, p = 0.470, Table 4).

**Table 4:** Influence of DC and CO on outcome parameters.

Outcome	n [mean age ± SD]	DC, n	CO, n	*p*
Survivors	296 [52.2 ± 19.5]	263	33	< 0.001
Deceased	299 [59.6 ± 19.3]	136	163	
Organ donation				
Deceased without donation	289 [60.7 ± 18.4]	129	160	0.194
Deceased with donation	10 [26.1 ± 15.5]	7	3	
Reason for no donation				
Preserved brain stem reflexes or inconclusive diagnostic of BD	90 [65.6 ± 13.7]	43	47	0.470
Other reasons	199 [58.6 ± 19.8]	86	113	

*SD* standard deviation, *DC* decompressive craniectomy, *CO* craniotomy.

A one-way MANOVA showed a statistically significant difference between utilized DBD on the combined dependent variables age and DC or CO (F [2, 296] = 17.625, p < 0.001, partial η^2^ = 0.106, Wilk’s Λ = 0.894). Post-hoc univariate ANOVA showed a statistically significant difference between the utilized DBD for age (F [1, 297] = 34.516, p < 0.001, partial η^2^ = 0.104), but not for DC or CO (F [1, 297] = 2.512, p = 0.114, partial η^2^ = 0.008).

### Proportion of DC and CO during the observation period

**Figure 2:**
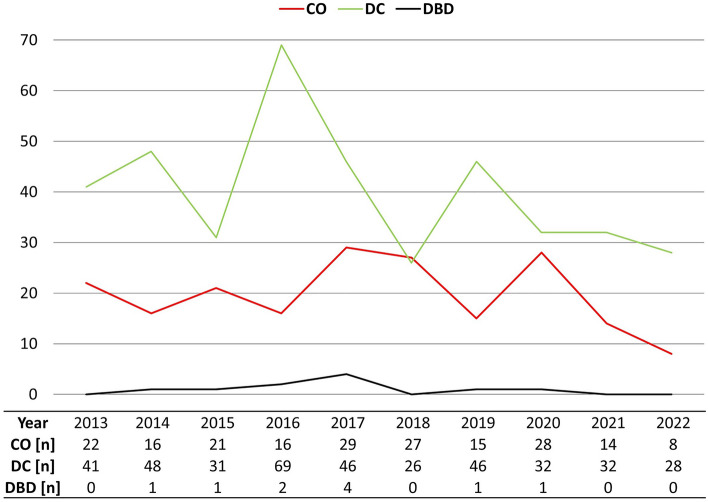
Identified patients per year. *CO* craniotomy, *DBD* donation after brain death, *DC* decompressive craniectomy.

Proportion of DC was between 49 and 81% during the observation period but did not show an increase over time (Fig. 2).

## Discussion

A total of 595 patients who underwent CO or DC after severe BI were identified between 2013 and 2022. The proportion of DC compared to CO was significantly higher in survivors than in deceased. 299 patients died during their hospital stay, and 10 DBD were utilized. Length of hospital stay, anticoagulatory medication and prevalence of comorbidities at the time of hospital admission did not differ between deceased after DC or CO. Preserved brain stem reflexes as a reason against utilization and the proportion of utilized DBD were not different between deceased after DC or CO. The proportion of DC compared to CO did not increase during the observation period. These results suggest that the diagnosis of BD in patients after DC or CO is generally low and the number of potential and utilized DBD are comparable in deceased after DC or CO.

Not surprisingly, patients with DC were significantly younger than patients with CO, as DC is a possible life-saving intervention, and indication to perform DC presumably increases with younger age^27,28^. This was also found in previous studies for DC after TBI^27,29^, AIS^30^, ICH^2,28^ or SAH^31^. It is also conceivable, that utilized DBD were significantly younger than the deceased without donation, presumably because medical contraindications are more common in older potential DBD^32^ and consent to donation tends to be higher in younger patients, especially after traumatic BI^33^.

Although this was not a primary aim of our study, results showed that the proportion of DC was significantly higher in survivors than in deceased, indicating a lowered mortality rate after DC compared to CO. Since we were not able to assess all information retrospectively, such as the severity of BI in each case, or the period between initial BI and DC, we cannot conclude with certainty from our results, that DC on its own improved patients’ outcome^19^. However, since more patients survived after DC, it can be assumed that DC had the potential to prevent death when considering the entire cohort studied.

Almost eighty percent of the deceased suffered from BI after TBI, AIS or ICH, whereas the proportion of DC was highest in patients with anoxic BI after cardiac arrest (albeit the total number of patients with anoxic BI (n = 5) was very low), followed by AIS and SAH. Notably, in this unselected cohort, patients with anoxic BI and SAH were younger than patients with TBI or AIS. Although broader evidence supports the use of DC in patients with TBI^5^ and AIS^7^ compared to DC after other causes of BI^4^, it is conceivable that in our cohort, the indication for DC was influenced to a considerable extent by the age of the patient and not only by the cause or severity of BI. Additionally, there is very limited literature on DC or CO after anoxic brain injury following cardiac arrest. A recent review describes interventions to improve outcomes for this group of patients but does not discuss neurosurgical intervention options in detail^34^. Patients with anoxic brain injury were the youngest in this study and the proportion of DC was the highest, suggesting that DC after anoxic brain injury may be an act of desperation in the absence of evidence.

Predominant reasons for a donation not being utilized in the deceased were refused consent and the impossibility to diagnose BD because of preserved brain stem reflexes. These results are in line with previous findings in Germany and elsewhere about reasons for a donation not utilized^18,32,35–37^.

Overall incidence of BD, and numbers of potential and utilized DBD was very low in our cohort of patients with severe BI, comparable to previous findings in patients after DC^22^ and to potential donor numbers in Germany^38–40^ and elsewhere^41^. Others reported a BD incidence of 7.3% in patients after DC^22^, which is comparable to our results (confirmed BD in 8 out of 136 patients with DC, which corresponds to 5.8%). Ten utilized DBD out of 299 deceased in our study correspond to 3.3% utilized DBD, what seems low at first sight, but represents an even higher rate of utilized DBD than the official numbers from the German Organ Procurement Organization about utilized DBD in Germany (1.4% utilized donors among all deceased in hospital with a diagnosed brain injury in 2022, although it should be mentioned, that these numbers include all deceased with brain injury, and not only deceased after neurosurgical procedures)^42^. This indicates that it is not DC or CO per se that are accountable for the low number of utilized DBD in this cohort, but that other reasons must have played a significant role. 33 patients became no DBD, because therapy was withdrawn before signs of BD established, presumably due to an inevitably poor prognosis and the possible assumption by clinical staff that patients may not develop signs of BD^22^. Additionally, 90 deceased became no donor because BD could not be confirmed due to preserved brain stem reflexes or inconclusive diagnostic of BD. Both groups of deceased would potentially become donors after cardiac death (DCD) after withdrawal of therapy^18^, but DCD is not possible in Germany due to legal regulations. It can be assumed, that a significant share of potential donors was thus lost because of these circumstances^38^. Refused consent precluded a donation in a further 112 cases.

We could not find a difference between DC and CO and the number of potential and utilized DBD in the cohort of the deceased. Interestingly, even more patients after DC became utilized DBD than after CO, although this finding was not statistically significant due to the overall low number of utilized DBD. As the indication for DC after severe BI is to prevent or treat an increase of ICP and thus prevent a vicious circle leading to BD, this result is unexpected at first sight. This circumstance likely reflects more severe BI in patients, where DC was performed, resulting in a more likely progression to BD. However, this assumption cannot be assured from the results, as severity of BI was not assessed in our study. Nevertheless, these findings are important for critical care and organ procurement staff. DC in patients with severe BI might indicate a more likely progression to BD and, in turn, into a utilized DBD, rather than a reason for prevention of a DBD in patients with an unfavorable prognosis. This is a relevant aspect, as being not considered by staff as a potential DBD was an important reason for DBD not utilized after DC in other studies^18,43^. Intensive care is probably withdrawn before patients with an inevitably poor prognosis develop signs of BD after DC. This may be because patients with DC are often not considered to qualify for BD development, as it is assumed that ICP does not reach critical levels causing loss of cerebral perfusion^17,22^. Critical care staff should therefore be aware to consider a potential DBD in patients with severe BI, regardless of the neurosurgical procedure. Additionally, preserved brain stem reflexes, as an indicator for the absence of BD, were not more common after DC than CO in our cohort, indicating, that the progression to BD in the deceased was independent of the neurosurgical procedure. The pathophysiological reasoning behind these findings was previously examined by Salih et al. They could show that loss of cerebral perfusion provides the key pathophysiological mechanism of BD after DC. Arterial blood pressure, dropping below a critical closing pressure was not modified by DC in their analysis. Consequently, even after DC, ICP may increase and reach levels critical enough to cause loss of cerebral perfusion^22,44^. In addition, cerebral ischemia can occur even if ICP and CPP are within the accepted thresholds^45,46^.

This study was a single-center retrospective analysis, resulting in several limitations. The practice patterns for performing DC might have varied greatly during the long period. We did not assess the surgical techniques used to perform DC (bifrontal craniectomy, unilateral or bilateral front-temporo-parietal craniectomy^47^), the initial severity of BI and the period between BI and DC or CO. We only included patients with DC or CO but not with other or no surgical therapy. We did not assess individual medical interventions to prevent or treat increased ICP. In addition, in cases where treatment was withdrawn due to refused consent, BD could have occurred later if treatment had been continued. All these factors might have an influence on patient outcomes. However, as the literature about accurate estimations of potential DBD is generally scarce and lack evidence^20,22^, our main goal was to evaluate, if DC and CO affect the progression to brain death and utilized donations differently in the cohort of patients with an unfavorable prognosis, regardless of the timing or type of the procedure or the patient’s condition. Since significantly more patients survived after DC than after CO, it can only be concluded that DC and CO had comparable effects on the progression to BD in the cohort of the deceased. Regarding the higher survival rate after DC, it must be assumed that DC was able to reduce BD when considering the entire study population.

In conclusion, these results add important information to the sparse literature on potential BD in patients after severe BI and the influence of different treatment modalities.

It was never the aim of our study to imply with our results that the choice of treatment should be influenced by considering the patient as a potential DBD. The choice of treatment is always made with the aim of doing what is best for the patient and respecting their wishes in terms of quality of life^16,48^. Nevertheless, in certain patients with an unfavorable prognosis, considering a potential DBD may be an important part of an end-of-life approach. Patients should not be deemed ineligible or unlikely to progress to BD based on the previous therapy. Future studies on outcomes after severe brain injury should include information on the incidence of BD to allow accurate estimation of potential DBD. This is necessary to respect a patient's possible wish for organ donation and to increase the number of utilized donations after brain death^20^.

## Methods

This retrospective study was performed in accordance with the Declaration of Helsinki. The need for informed consent was waived by the local Ethics Committee of the University of Münster due to the retrospective analysis of routinely collected patient data and the study protocol was approved on November 22, 2019 (file number 2019-389-f-S). All patients treated at the University Hospital Münster between January 2013 and December 2022 who underwent CO or DC to prevent or treat consequential damage after severe BI were retrospectively included, regardless of the individual indication for either procedure in each case (Fig. 1). These patients were cared for in the same intensive care units, with a consistent management protocol. These protocols, which are described in our hospital's standard operating procedures, include both general instructions for neurosurgical patients (daily evaluation of the Glascow Coma Scale and the Richmond Agitation-Sedation Scale, continuous monitoring of intracranial pressure if available, definition of target values for arterial blood pressure, laboratory blood coagulation values, use of anticoagulants and interventions to decrease intracranial pressure, e.g. by the use of mannitol, venting of cerebrospinal fluid through the ventricular drain, and/or controlled hyperventilation) and specific instructions depending on the underlying disease (e.g. enteral nimodipine and daily transcranial Doppler ultrasonography in patients with non-traumatic subarachnoid hemorrhage).

Identification of patients was based on the German Operation and Procedure Code (OPS-Code, the official classification for coding operations and procedures in Germany^49^). All patients with the OPS-Code 5-012 (this code is to be used for an isolated CO (OPS-Code 5-012.1/x) or DC (OPS-Code 5-012.0), whereas a CO or DC as a surgical access route is coded separately) were included. The in-house medical controlling department provided the data.

In the next step, all patients who survived (reason for discharge from the hospital was not “death”) were excluded and the deceased were further analyzed. The medical record files of the deceased were screened for anticoagulatory medication and comorbidities at the time of hospital admission, hospital length of stay, the cause of BI [traumatic brain injury (TBI), non-traumatic subarachnoid hemorrhage (SAH), spontaneous intracerebral hemorrhage (ICH), acute ischemic stroke (AIS), brain tumor (BT), infections of the central nervous system (CNS) and anoxic BI after cardiac arrest, respectively]. The number of utilized DBD or the reason for a donation not utilized (refused consent, preserved brain stem reflexes/inconclusive diagnostic of BD, medical contraindications, therapeutic limitations according to patients’ will) was recorded. Finally, to analyze the possibility of DBD, deceased with CO and DC were compared regarding the number of utilized DBD and the reasons against utilization, respectively.

### Statistical methods

Statistical analysis was performed using SPSS 28 (IBM, USA). Parameters were expressed as mean ± standard deviation. The χ^2^-test or Fishers exact test (in case of small-sized samples) were used to check whether variables were independent and the t-test for independent samples for group comparison. A one-way multivariate analysis of variance (MANOVA) was used to compare DC or CO and predefined combined dependent variables (length of hospital stay, preexisting use of anticoagulants and comorbidities) and for the number of utilized donations and predefined combined dependent variables (age and DC or CO). Post-hoc univariate ANOVAs were conducted for every dependent variable. A statistical significance was assumed at p ≤ 0.05.

## Data Availability

The datasets generated and analyzed during the study are available from the corresponding author on reasonable request. Requests to access these datasets should be directed to jan.englbrecht@ukmuenster.de.
